# Bloom’s taxonomy—Can evidence-based teaching improve junior medical officers’ knowledge of the mental health and guardianship acts?

**DOI:** 10.1177/10398562241260170

**Published:** 2024-06-14

**Authors:** Trent Koessler, Warren Kealy-Bateman

**Affiliations:** Psychiatry Registrar, 58416 Western NSW Local Health District, Dubbo, NSW, Australia; School of Medicine, 8691 University of Wollongong, Wollongong, NSW, Australia;; School of Rural Health, University of Sydney, Dubbo, NSW, Australia; and; Clinical Director Mental Health Drug and Alcohol, Dubbo and Regions, 58416 Western NSW Local Health District, Dubbo, NSW, Australia

**Keywords:** Mental health act, guardianship act, education, restrictive care, Bloom’s taxonomy

## Abstract

**Objective:**

To determine whether a brief educational intervention for Junior Medical Officers (JMOs), using teaching methods aimed at achieving higher outcomes on Bloom’s Taxonomy, significantly improved participant confidence and knowledge in decision making about restrictive care.

**Method:**

JMOs received a teaching session on restrictive medical and mental health care. Groups were randomly assigned to either sessions including a component of modern pedagogical interventions (Think-Pair-Share and SNAPPS), or sessions including a control period focusing on reviewing a condensed summary of relevant information. Pre- and post-intervention measures were recorded for subjective self-ratings of confidence and scores on standardized clinically relevant extended matching questions (EMQs).

**Results:**

There was no difference in subjective confidence improvement between groups; however, the group receiving the modern pedagogical intervention demonstrated significantly greater objective performance on knowledge-based EMQs.

**Conclusions:**

A brief modern pedagogical intervention using interactive teaching methods shows promise for improving knowledge of restrictive care and the Mental Health and Guardianship Acts. In the control group, similarly increased confidence in knowledge did not equate to increased competence on a knowledge assessment. Refurbishing educational interventions presents opportunities for improving clinical outcomes and engaging junior doctors in psychiatry.

New medical graduates encounter numerous challenges as they are expected to acquire multiple competencies across various specialties and domains. One such competency involves working with state and territory specific restrictive care legislation, such as the Guardianship Act 1987 (NSW)^1^ and Mental Health Act 2007 (NSW).^2^ These permit the restriction of patient freedoms under certain circumstances. The respective purview of these Acts can be nuanced. Junior medical officers (JMOs) make critical decisions involving this legislation with significant implications for patient liberty, safety, and wellbeing. This likely depends on restrictive care skills learned throughout their training relevant to both Acts.

Research indicates that real-world use of this legislation is error prone. Studies demonstrate^3^ that even in a tertiary setting over 10% of mental health medical certificates fail to meet the requirements of the Mental Health Act (MHA), with error rates increasing to over 30% for certificates completed by emergency department staff. Other studies report widespread errors and invalidity^4^ particularly for generalist medical practitioners compared to psychiatry staff.^5^ Incorrectly completed MHA documentation could potentially invalidate detention, raising human rights concerns.

## Medical education interventions

There are a lack of educational interventions addressing deficits in the proper use of restrictive care.^6^ Wand and colleagues^7^ developed a pilot workshop for psychiatry trainees that included teaching on restrictive care using presentations and interactive discussion. Self-rated knowledge and confidence in duty of care improved, but not knowledge or application and communication of the MHA. The half-day training in this intervention may also be difficult to implement for many cohorts of JMOs.

**Figure 1.**
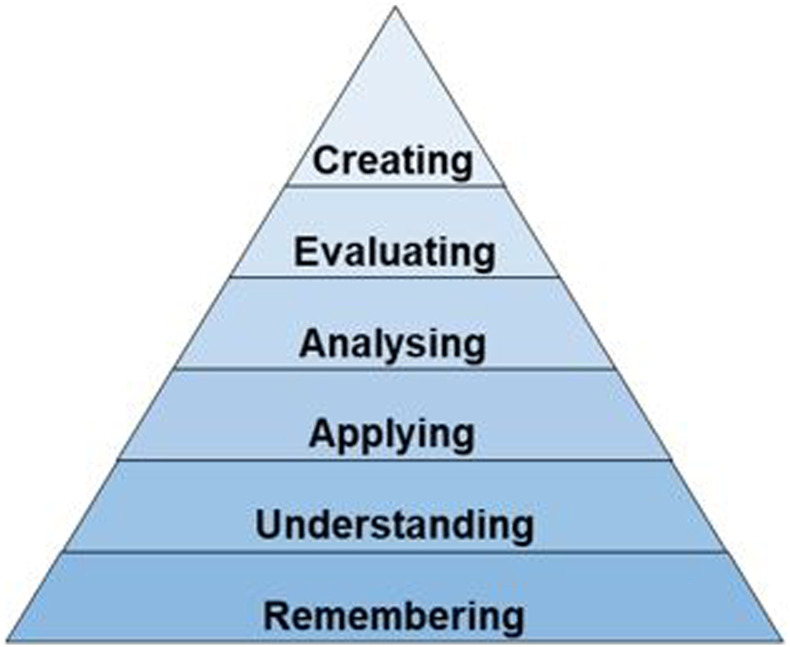
Revised Bloom’s Taxonomy.

While medical graduates learn about restrictive care as part of medical school and graduate curricula, there remains a high rate of misuse and misunderstanding. This suggests a change in educational strategy may be warranted. The traditional teaching formats generally used may address the two lowest aspects of the Revised Bloom’s Taxonomy^8^ (see Figure 1)—remembering and understanding—but leave the more complex cognitive skills such as applying, analyzing, evaluating, and creating, unaddressed. Furthermore, beyond improving competence, engaging JMOs in higher-order thinking presents an opportunity to encourage learners to consider future psychiatric practice by empowering them to use foundational skills.

## SNAPPS and Think-Pair-Share

Evidence-based educational interventions, such as SNAPPS^9^ and Think-Pair-Share,^10^ offer a potential solution to these limitations by emphasizing active engagement, collaboration, and the practice of higher-order cognitive skills. SNAPPS is a learning format that involves working through case vignettes using structured, collaborative learning, engaging learners in the steps summarized in Table 1. Think-Pair-Share, on the other hand, encourages independent thinking on a topic, discussion with peers, and presenting integrative responses in a group. Learners can be primed about the teaching format using a flipped classroom format,^11^ which requires learners to prepare before a teaching session to facilitate active engagement during face-to-face sessions.

**Table 1.** SNAPPS Educational intervention

S	Summarize briefly the history and findings
N	Narrow the differential to two or three relevant possibilities
A	Analyze the differential comparing and contrasting the possibilities
P	Probe the preceptor by asking questions about uncertainties, difficulties, or alternative approaches
P	Plan management for the patient’s issues
S	Select a case-related issue for self-directed learning

This project aimed to determine if a modern pedagogical intervention (MPI) for JMOs improved: (1) participant confidence in dealing with restrictive care issues and (2) knowledge of restrictive care decision-making, as reflected in an objective change in doctors’ ability to answer questions relating to restrictive care scenarios.

## Methods

This study was deemed to be a quality improvement project by the local ethics processes of Western NSW Local Health District (WNSWLHD, 2022/ETH01019). The project took place between 2022 and 2023, during four pre-existing teaching sessions for JMO postgraduate years one and two in WNSWLHD. The content of the session focused on restrictive care, using anorexia nervosa as a complex example relevant to several rotations undertaken by junior doctors. Recruitment was via normal attendance to teaching, preceded by emails inviting voluntary participation through data submission (via paper forms). Attendance did not mandate data provision, although in practice all learners contributed.

The project employed a 2x2 within-subjects design, with factors of intervention type and time with respect to intervention. Learner groups were randomly allocated to a one-hour session that either utilized a new MPI teaching format involving SNAPPS and Think-Pair-Share, or an active control intervention with an equal amount of time allotted to review a condensed summary of relevant information (including a one-page algorithm on restrictive care legislation,^12^ two NSW Civil and Administrative Tribunal (NCAT) factsheets on restrictive care,^13,14^ and a copy of section 37 of the Guardianship Act). Interactive segments encouraged JMOs to apply the discussed principles to other clinical scenarios such as mania or delirium. Pre-reading material on teaching methods was distributed to all learners by administrative staff.

Intervention effectiveness was measured via comparison of pre- and post-intervention assessments of both groups. To assess knowledge, learners were randomly allocated four extended matching questions (EMQs, see Table 2 for an example) from one of two fictious clinical scenarios, with the alternate set used post-session. The order of questions within a set was also randomized.

**Table 2.** Example of an extended matching question (EMQ)

EMQ - Select the most appropriate action for each scenario
1.Schedule patient as mentally disordered under s19 of the Mental Health Act (2007)
2.Schedule patient as mentally ill under s19 of the Mental Health Act (2007)
3.Detain and treat patient under the Guardianship Act (1987)
4.Discharge patient
5.Do nothing
# You are a senior resident working after hours. You are called to review patient X who is agitated and aggressive. All attempts at verbal de-escalation and less restrictive practices have failed.

	Question	1	2	3	4	5
1	You think that patient X will walk in front of a car if not given pharmacological sedation	☐	☐	☐	☐	☐

**Figure 2.**
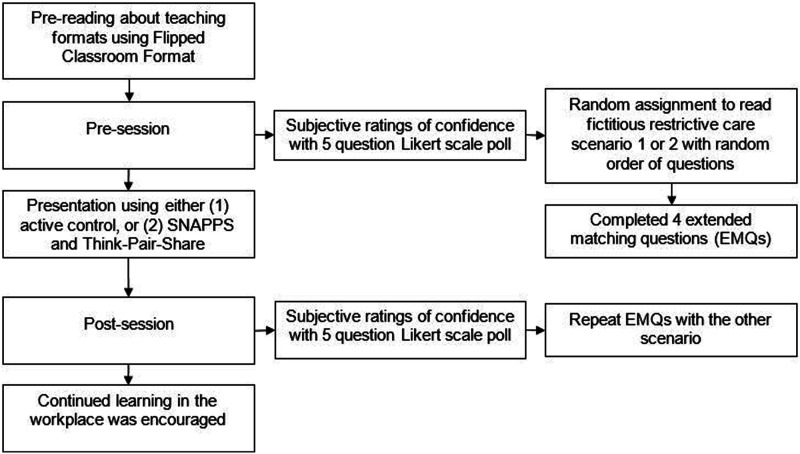
Map of the educational intervention including testing and delivery.

Confidence was assessed with a 5-point Likert scale ranging from “Strongly disagree” to “Strongly Agree” (questions listed in Table 3). See Figure 2 for a pictorial representation of intervention delivery and testing. Difference scores for both sets of data were compared non-parametrically with a Wilcoxon rank sum test.

**Table 3.** Difference between groups for pre- and post-session Likert score change (*n* = 32)

Question	Mean difference (SE)	Wilcoxon W	Z-score	*p*-value
I have received sufficient education and training on restrictive care in medicine to confidently apply them	0.557 (0.270)	233.000	−1.975	0.048
I know how to correctly complete mental health Act documents	−0.345 (0.324)	223.500	−0.954	0.340
I know the requirements one has to meet in order to be considered mentally ill or mentally disordered	0.577 (0.330)	248.000	−1.315	0.189
I know the difference between when to apply the Mental Health Act (2007) and when to apply the Guardianship Act (1987)	0.639 (0.246)	222.500	−2.450	0.014
I can confidently assess and manage a situation in which restrictive care principles may be relevant	0.400 (0.239)	244.500	−1.491	0.136

p/5 < .05 (Bonferroni correction).

## Results

Thirty-two JMOs attended (MPI *n* = 15; active control *n* = 17), none more than once. Data were combined across sites. Prior to the intervention, participants on average selected either strongly disagree or disagree for all statements of confidence. Confidence ratings for all questions improved after the intervention for both groups. However, there was no significant difference in confidence improvement between the MPI and control group (see Table 3).

In contrast to this finding, for EMQ performance learners who received the MPI session showed significantly greater scores than the control group (see Table 4). Post-hoc analysis of within-subjects differences with a Wilcoxon signed-rank test revealed only the MPI group (Z = −2.944; *p* = 0.003), not the control group (Z = −0.465, *p* = 0.642) significantly improved their EMQ scores after the session, suggesting improvement in objectively measured knowledge.

**Table 4.** Difference between groups for pre- and post-session Extended Matching Question scores (*n* = 32)

	Mean difference (SE)	Wilcoxon W	Z-score	*p*-value
Extended matching question score	0.882 (0.365)	223.00	−2.273	0.023*

*p < 0.05.

## Qualitative results

Seven qualitative feedback responses were received—all for the MPI group (see Table 5). All seven respondents rated the relevance of the session as “Excellent” on a 5-point Likert scale ranging from “Excellent” to “Terrible.”

**Table 5.** Qualitative feedback from the learners

Qualitative comments included
“Very good presentation”
“Very good”
“Really improved confidence in skills of coercive care. Interactive approach really helped solidify learning”
“Engaging”
“This was excellent content. I feel much more confident with section 19, form 1 and Guardianship Act”
“Excellent”

Suggestions for improvement were predominately related to requests for further teaching
“MSE”
“NA”
“Suicide prevention and risk assessment”
“ [blank]”
“Perhaps the MSE when psych give us more teaching”
“Excellent”

## Discussion

Ideally, JMOs should have basic competence in restrictive care and be able to clinically navigate related issues. This involves situations that carry significant risks, are time-sensitive, affects the lived experience of the person and their loved ones, and includes heightened emotional factors impacting judgment. JMOs may be asked to complete associated documentation, yet education is lacking. Consistent with previous research, prior to intervention JMOs lacked confidence and competence in their knowledge around restrictive care decision making.

This study showed that a realistically achievable educational intervention, lasting just 1 hour, during a normal teaching slot, improved JMO knowledge in the topic. Confidence increased similarly in both groups. For the active control group, this did not correlate with EMQ performance improvements, suggesting possibly inflated self-assessment.

The strategic use of evidence-based tools to target higher learning outcomes on Bloom’s taxonomy via MPI, rather than the specific content of the presentation, is suspected to have contributed to positive outcomes. The qualitative feedback received was consistent with this, suggesting that the session engaged participants, who praised the interactive approach. Indeed, only the MPI learners provided qualitative feedback. The use of evidence-based educational techniques, such as Think-Pair-Share and SNAPPS, highlight the potential for broader utility in medical education and beyond. These interactive teaching strategies are effective, adaptable, and easily implementable, even for participants unfamiliar with their use.

## Limitations

A relatively small sample size (*n* = 32) limits the statistical power of this study. It is also of interest whether these findings extend to other cohorts and educational topics.

## Conclusion

These findings both align with and expand upon previous research on educational interventions teaching restrictive care. While earlier studies have also shown improvements in confidence after educational interventions, this study emphasizes the value of deliberately targeting more taxonomically sophisticated learning strategies with methods such as Think-Pair-Share and SNAPPS to improve performance.

Further research is warranted to investigate the specific variables that can optimize the delivery of these educational interventions, as well as their potential to reduce incidents such as incorrectly completed mental health certificates. Follow-up to determine whether SNAPPS encouraged learning beyond the workshop as intended would also be of interest. This study highlights the potential for implementing similar interventions in diverse settings and at various stages of medical training. More effective engagement with JMOs also presents potential opportunities for broadening the appeal of psychiatry training.
